# The Genetic Variation of *SORCS1* Is Associated with Late-Onset Alzheimer’s Disease in Chinese Han Population

**DOI:** 10.1371/journal.pone.0063621

**Published:** 2013-05-20

**Authors:** Wei Xu, Jun Xu, Ying Wang, Huidong Tang, Yulei Deng, Rujing Ren, Gang Wang, Wenquan Niu, Jianfang Ma, Yiwen Wu, Jialin Zheng, Shengdi Chen, Jianqing Ding

**Affiliations:** 1 Department of Neurology and Institute of Neurology, Rui Jin Hospital, School of Medicine, Shanghai Jiao Tong University, Shanghai, China; 2 Department of Neurology, Nanjing Medical University Affiliated Nanjing Brain Hospital, Nanjing, Jiangsu, China; 3 State Key Laboratory of Medical Genomics, Rui Jin Hospital, School of Medicine, Shanghai Jiao Tong University, Shanghai, China; 4 Pharmacology and Experimental Neuroscience and Pathology and Microbiology, University of Nebraska Medical Center, Omaha, Nebraska, United States of America; 5 Lab of Neurodegenerative Diseases and Key Laboratory of Stem Cell Biology, Institute of Health Science, Shanghai Institutes of Biological Sciences, Chinese Academy of Science and School of Medicine, Shanghai Jiao Tong University, Shanghai, China; Stanford University School of Medicine, United States of America

## Abstract

The variations of *SORCS1* gene may play potential key roles in late-onset Alzheimer’s disease (LOAD). To evaluate the relationship between the polymorphism of *SORCS1* gene and LOAD in the ethnic Han Chinese, we conducted a case–control study to investigate the association between the single-nucleotide polymorphisms (SNPs) in intron 1 of *SORCS1* and LOAD in Chinese Han population. Six reported SNPs in intron 1 of *SORCS1* were analyzed by Snapshot, genotyping and haplotyping in 236 Chinese LOAD cases and 233 matched controls. The significant differences in frequencies of two SNPs (rs10884402, rs950809) were found between the two groups. In addition, haplotype analyses revealed that, in the LOAD group, the frequency of haplotypes C-C-G-T-C (alleles in order of rs17277986, rs6584777, rs10884402, rs7078098, rs950809 polymorphisms) were significantly higher (Psim<0.0001) while haplotype C-C-A-T-C, C-C-A-C-C, T-T-A-C-C were significantly lower (Psim<0.0001). Our data suggested that the genetic variation of the rs10884402 and rs950809 in intron 1 of *SORCS1* was associated with the late-onset AD in the Chinese Han population.

## Introduction

Alzheimer’s disease is the most common form of dementia, which is characterized by senile plaques, neurofibrillary tangles and neuron loss [1], [2]. In the early-onset family AD (EOFAD), three genes, amyloid precursor protein (APP), presenilin 1 (PS1), or presenilin 2 (PS2) [3], [4] were demonstrated to directly influence Aβ metabolism. In contrast to EOFAD, several risk genes such as apolipoprotein E (APOE), EPHA1, CD33 and MS 4A 6A [5], [6], [7] are involved in the pathogenesis and development of late-onset Alzheimer’s disease (LOAD), in which APOE is the most notable. APOE ε4 allele may account for nearly 50% of the genetic risk in LOAD [8], [9].

Although the pathogenetic mechanisms of AD are undetermined, the APP processing and Aβ generation have been proven to be crucial in the pathogenesis of AD [10], [11]. Extracellular accumulation of the amyloid-β (Aβ) peptides leads to senile plaques formation. Sorting mechanisms that lead the colocalization of APP, β- secretases and γ-secretases in the same intracellular compartment may play an important part in Aβ generation in AD [12], [13], [14]. Sortilin-related VPS10 domain containing receptor 1 (*SorCS1*), which maps to chromosome 10q23–25, belongs to Vps10p-domain sorting receptor family [15], [16], [17]. SorCS1 is prominently expressed in the nervous system and may be important for neuronal activities [15], [18]. SorCS1 was reported that it could influence APP processing and modulate Aβ metabolism [19], [20]. Overexpression of SorCS1 might lead to the reduction of γ-secretase activity and Aβ levels. Oppositely, suppression of SorCS1 increased γ-secretase processing of APP and the levels of Aβ [20]. In addition, one genome-wide association study (GWAS) in French has identified *SORCS1* as a candidate gene for AD [21]. All these suggested that *SORCS1* were associated with the prevalence of AD.

Based on the result reported by Reitz and his colleagues which indicated several SNPs in intron 1 of *SORCS1* were genetic associated with LOAD and memory retention [20], [22], we conducted a case–control study (n = 469) to determine the prevalence of six reported SNPs (rs17277986, rs6584777, rs12251340, rs10884402, rs7078098 and rs950809) in patients with LOAD in Chinese Han population of mainland, trying to explore the genetic association between the polymorphism of *SORCS1* and LOAD. As APOEε4 allele and the history of type-2 diabetes mellitus were confirmed worldwide as important factors to LOAD, we further analyzed the relationship between these factors and SNPs in LOAD.

## Methods

### Subjects

Our study included 236 sporadic LOAD and 233 healthy controls of Chinese Han ethnicity. All patients, which were enrolled from the outpatient clinic at the Department of Neurology, Ruijin Hospital affiliated to Shanghai Jiaotong University School of Medicine and were evaluated by a experienced neurologist and a psychiatrist, had a clinical diagnosis of possible or probable AD according to the Alzheimer’s Criteria of National Institute of Neurological and Communicative Disorders and Stroke and the Alzheimer’s Disease and Related Disorders Association (NINCDS-ADRDA) [23]. All control subjects were recruited from the epidemiological investigation of Shanghai, which were matched for age, gender, and ethnic background. All subjects were unrelated Chinese Han and had no family history of AD. The average age of AD group was 72.28±7.87 years old (Mean age at onset) with the average MMSE score 15.96±5.99, 50.8% were male AD. And the average age of control was 72.88±7.30 years old (Mean age at examination) with the average MMSE score 28.35±1.42, male accounted for 58.3%. The study was approved and authorized by the Research Ethics Committee, Rui Jin Hospital affiliated to School of Medicine, Shanghai Jiao Tong University, Shanghai, China. All participants were fully informed, and had signed a formally written consent.

### Genotyping

DNA was isolated from peripheral blood through standardized phenol/chlorine extraction method. Genotyping analysis of APOE was performed as previously described [24]. The *SORCS1* SNPs (rs17277986, rs6584777, rs12251340, rs10884402, rs7078098 and rs950809) were genotyped using the method of SNaPshot, which was based on the dideoxy single-base extension of an unlabeled oligonucleotide primer (or primers), with technical support from the Shanghai Southgene Technology Co. LTD.

All amplification primers were synthesized by standard phosphoramidite chemistry (Sangon Biotech). The primers and probe sequences which were used are summarized in Table 1. The amplification of the target fragment was carried out on a PCR Amplifier (MJ Research PT-100) in a total volume of 10 µl containing ∼20 ng of DNA, 0.4 µM each of the primers, 0.3 mM dNTP (Generay Biotech), 0.25 U HotStarTaq DNA Polymerase (QIAGEN). The final concentration of Mg^2+^ in the reaction mixture was adjusted to 3.5 mM. The cycle conditions were as following: denaturation of the template DNA for 1 cycle of 95°C for 5 mins; amplification of the target fragment for 45 cycles of 95°C for 30 s, 60°C for 60 s, and 72°C for 180 s. The PCR products were electrophoresed on 2% agarose gel and visualized under UV light. 2 U Shrimp Alkaline Phosphatase (USB) and 2 U Exonuclease I (Epicentre) was used to purify the target fragment. The mixture was incubated at 37°C for 1 hour and then was incubated at 75°C for 15 mins to inactivate the enzymes. All the primers to be used for SNaPshot reaction should be premixed to reach a final concentration of 0.2 µM. The total volume of SNaPshot PCR mixture was 5 µl, containing 1 µL SNaPshot Multiplex Ready Reaction Mix (ABI), 2 µL Pooled PCR products, 1 µL Pooled SNaPshot primers and 1 µL deionized water. The cycle conditions for SNaPshot were as following: denaturation of the template for 1 cycle of 95°C for 10 s; amplification of the target fragment for 25 cycles of 95°C for 10 s, 50°C for 5 s, and 60°C for 30 s. Add 0.5 Unit of Shrimp Alkaline Phosphatase (USB) to the reaction mixture, mix thoroughly, and incubated at 37°C for 1 hour. The enzyme was deactivated by incubating the mixture at 75°C for 15 mins. Dilute 0.5 µL of SNaPshot product and 0.25 µL of GeneScan-120 LIZ (ABI) in 9.25 µL of Hi-Di formamide (ABI), vortex briefly and quick spin, then denature the samples by placing them at 95°C for 5 minutes. Electrophoresis was performed on the ABI PRISM 3730 DNA Analyzer according to the manufacturer’s instructions.

**Table 1:** Primer design.

SNP ID		Primer Name	Sequence	TM(°C)
rs17277986	The PCR Primer	rs17277986-L	5′-TCAGTTCTCCCATTTGTTGCT-3′	59.73
[C/T]		rs17277986-R	5′-AGGCTCTTGGAAGGCATTTT-3′	60.21
	The anchor probe	rs17277986-SNP2	5′-ttttttttttCTCAGATTCCAAGAATTATTCAGC-3′	57.14
rs6584777	The PCR Primer	rs6584777-L	5′-CAGAGTGTGATCCCATCTCAA-3′	58.70
[A/G] and		rs6584777-R	5′-CTCCACCATGTGGAACTGTG-3′	60.00
rs12251340 [G/T]	The anchor probe	rs6584777-SNP	5′-ttttttttttTAACTCCTGATATCCAAGTTTGTATTC-3′	57.03
		rs12251340-SNP	5′-CACCATGTGGAACTGTGAGT-3′	55.17
rs10884402	The PCR Primer	rs10884402-L	5′-TGCCTGTACAACGAGCTCAC-3′	60.06
[A/G]		rs10884402-R	5′-AGGTTCCCCTTTGCTGTTCT-3′	60.11
	The anchor probe	rs10884402-SNP	5′-tttttttGCCAGCAGGAAAGAGAATGT-3′	57.92
rs7078098	The PCR Primer	rs7078098-L	5′-ACTCCTGATGCTCTGGGAGA-3′	59.94
[C/T]		rs7078098-R	5′-AGGGTGCTTCCAGATGTGAC-3′	60.12
	The anchor probe	rs7078098-SNP	5′-tttttttttttttttttTGATGCTGATTAACAGTTTTCCC-3′	58.97
rs950809	The PCR Primer	rs950809-L	5′-CATTGACAGGCAAAGCAAGT-3′	58.93
[C/T]		rs950809-R	5′-GCATGGTTCTCTTTGGAGGA-3′	60.20
	The anchor probe	rs950809-SNPr	5′-tttttttttttttttttttttTTGTAGGTGATGTTTGCAATCAGT-3′	59.41
		rs950809-SNP2	5′-ttttttttttttttttttttttttttttGATGAGGCATAGGGCTCACT-3′	57.74

### Statistical Analysis

Statistical calculations were done using SAS v.9.1.3 (Institute Inc., Cary, NC). Means of continuous variables were compared by unpaired t-test. The χ2 test or Fisher's exact test was used to assess the goodness-of-fit between the observed allele frequencies and the expected counterparts by Hardy–Weinberg equilibrium and to evaluate the differences in genotype and allele distributions between cases and controls. Each genotype was assessed by logistic regression analysis assuming additive, dominant and recessive modes of inheritance, respectively. A two-tailed P<0.05 was accepted as statistically significant.

The linkage disequilibrium patterns were identified in all samples by Haploview v.4.0 available at www.sourceforge.net. The linkage disequilibrium coefficients were shown as D' on the basis of 4 gamete color scheme. Traditionally, a haplotype was defined as a combination of multiple alleles in a chromosome. This was because alleles on the same chromosome were in the close proximity and might interact with each other. The haplo.em program was used to estimate the haplotype frequencies for the polymorphisms. This program estimated maximum likelihood of haplotype probability using the progressive insertion algorithm that progressively inserts batches of loci into haplotypes of growing lengths. The haplo.cc and haplo.glm were employed to calculate crude and adjusted odds ratios (ORs) and 95% confidence intervals (CIs) for each haplotype, respectively. These two approaches were based on a generalized linear model, and computed the regression of a trait on haplotypes and other covariates [25], [26]. Furthermore, the haplo.score was used to model an individual phenotype as a function of each inferred haplotype, weighed by their estimated probability, to account for haplotype ambiguity. It was based on score statistics, which provided both global tests and haplotype specific tests [27]. Simulated P (Psim) values were obtained from 1000 replicates. The haplo.em, haplo.glm and haplo.score were implemented in the program Haplo.stats software (version 1.4.0) developed by the R language (http://www.r-project.org).

## Results

The baseline characteristics between patients with Alzheimer’s disease and healthy controls were compared. No statistical differences were observed for age and gender between patients and controls (P>0.05). But significantly lower MMSE score was found in LOAD patients compared to the controls (P<0.001). Distributions of the APOE polymorphisms in both AD patients and controls were as expected.

### Single-point Association Analysis

There were no deviations from Hardy–Weinberg equilibrium for all studied polymorphisms in LOAD and controls (P>0.05). Three polymorphisms in SORCS1 did not reach significant differences in the genotype or allele frequencies in the total sample (genotype: rs17277986 P = 0.923, rs6584777 P = 0.982, rs7078098 P = 0.325; Allele: rs17277986 P = 0.719, rs6584777 P = 0.863, rs7078098 P = 0.207). rs10884402 polymorphism was demonstrated to have significant differences in both genotype and allele frequencies between the two groups in the total sample (genotype P = 0.0001; allele P = 0.0004). rs950809 polymorphism showed an edge difference in the genotype frequencies (P = 0.036) but no differences in the allele frequencies (P = 0.79). The results of three genetic modes of inheritance for the six studied genotypic polymorphisms in *SORCS1*, which were assessed by logistic regression analysis, were also shown in Table 2. rs10884402 showed significant differences in additive mode (OR = 0.63, 95% CI (0.49, 0.82), P<0.001) and recessive mode (OR = 0.33, 95% CI (0.2, 0.57), P<0.001) (Table 2). Since the genotype and allele frequencies of rs12251340 were completely all the same between the patients and the controls, we did not conduct the further analysis and discussion. When data were stratified by the history of type-2 diabetes mellitus or the severity of AD, no significant difference was observed (data not show). When data were stratified by ApoEε4, the significant differences of rs10884402 polymorphism were observed in both genotype and allele frequencies between the patients and controls in the ApoEε4 (−) population (genotype P = 0.008; allele P = 0.003) (Table 3). When data were stratified by gender, the significant differences for rs17277986 and rs6584777 were shown between male and female AD patients (genotype P = 0.007; allele P = 0.01) (Table 4). However, the further analysis did not find the significant difference between the male AD patients and the male controls or the female AD patients and the female controls (Table 5). The difference between overall male and female (genotype P = 0.003; allele P<0.0001) (Table 5) indicated the differences between male and female AD patients might be only related to the gender instead of disease. The other three SNPs showed no difference either between male and female AD patients (Table 4). These results suggested there were no gender association for all five SNPs.

**Table 2:** Genotype and allele frequencies for rs17277986, rs6584777, rs12251340, rs10884402, rs7078098 and rs950809 SNPs and three genetic modes of inheritance for the five studied polymorphisms in SORCS1 gene.

SNP ID	Group	n	Genotype frequency (%)			P-value	MAF	P-value	Models	OR; 95% CI; P^a^
			CC	CT	TT		T		Additive	0.93(0.64,1.36),0.71
rs17277986	AD	236	173(73.3)	60(25.4)	3(1.3)	0.922	0.14	0.719	Dominant	0.92(0.6,1.38),0.69
	Control	233	167(71.7)	63(27)	3(1.3)		0.15		Recessive	0.99(0.2,4.94),0.99
			CC	CT	TT		T		Additive	0.97(0.66,1.41),0.86
rs6584777	AD	236	173(73.3)	60(25.4)	3(1.3)	0.974	0.14	0.863	Dominant	0.96(0.64,1.45),0.85
	Control	233	169(72.5)	61(26.2)	3(1.3)		0.14		Recessive	0.99(0.2,4.94),0.99
			GG	GT	TT		T		Additive	NA
rs12251340	AD	236	236(100)	0(0)	0(0)	NA	0	NA	Dominant	NA
	Control	233	233(100)	0(0)	0(0)		0		Recessive	NA
			GG	GA	AA		A		Additive	0.63(0.49,0.82),<0.001
rs10884402	AD	236	102(43.2)	112(47.5)	22(9.3)	0.0001	0.33	0.0004	Dominant	0.7(0.48,1.02),0.06
	Control	233	81(34.8)	97(41.6)	55(23.6)		0.44		Recessive	0.33(0.2,0.57),<0.001
			TT	TC	CC		C		Additive	1.2 (0.91,1.58),0.208
rs7078098	AD	236	117(49.6)	101(42.9)	18(7.6)	0.325	0.29	0.207	Dominant	1.16 (0.81,1.66),0.428
	Control	233	107(45.9)	99(42.5)	27(11.6)		0.33		Recessive	1.59 (0.85,2.97),0.148
			CC	CT	TT		T		Additive	1.04(0.79,1.36),0.79
rs950809	AD	236	87(36.9)	126(53.4)	23(9.7)	0.036	0.36	0.79	Dominant	1.31(0.9,1.9),0.15
	Control	233	101(43.3)	98(42.1)	34(14.6)		0.36		Recessive	0.63(0.36,1.11),0.11

Abbreviations: OR, odds ratio; 95% CI, 95% confidence interval; MAF, minor allele frequency; NA, not available.

Genotypes and alleles are expressed as number (percentage). P values were calculated by χ^2^ test 3×2 contingency table for genotype distribution and 2×2 contingency table for allele distribution.

a Adjusting for age and gender.

**Table 3:** Genotype and allele frequencies for the five studied polymorphisms in SORCS1 gene in Alzheimer’s patients and controls stratified by ApoE carrier status.

SNP ID		Group	n	Genotype frequency (%)			P-value	MAF	P-value
				CC	CT	TT		T	
	ApoEε4(−)	AD	130	94(72.3)	34(26.2)	2(1.5)	0.899	0.15	0.94
rs17277986		control	189	136(72)	50(26.4)	3(1.6)		0.15	
	ApoEε4(+)	AD	106	79(74.5)	26(24.5)	1(1)	0.675	0.13	0.72
		control	44	31(70.5)	13(29.5)	0(0)		0.15	
				CC	CT	TT		T	
	ApoEε4(−)	AD	130	94(72.3)	34(36.2)	2(1.5)	0.988	0.15	0.91
rs6584777		control	189	138(73)	48(25.4)	3(1.6))		0.14	
	ApoEε4(+)	AD	106	79(74.5)	26(24.5)	1(1)	0.675	0.13	0.72
		control	44	31(70.5)	13(29.5)	0(0)		0.15	
				GG	GA	AA		A	
	ApoEε4(−)	AD	130	57(43.9)	58(44.6)	15(11.5)	0.008	0.34	0.003
rs10884402		control	189	65(34.4)	76(40.2)	48(25.4)		0.46	
	ApoEε4(+)	AD	106	45(42.5)	54(50.9)	7(6.6)	0.199	0.32	0.2
		control	44	16(36.4)	21(47.7)	7(15.9)		0.4	
				TT	TC	CC		C	
	ApoEε4(−)	AD	130	68(52.3)	50(38.5)	12(9.2)	0.515	0.28	0.24
rs7078098		control	189	87(46)	80(42.3)	22(11.7)		0.33	
	ApoEε4(+)	AD	106	49(46.2)	51(48.1)	6(5.7)	0.462	0.3	0.58
		control	44	20(45.4)	19(43.2)	5(11.4)		0.33	
				CC	CT	TT		T	
	ApoEε4(−)	AD	130	47(36.2)	68(52.3)	15(11.5)	0.202	0.38	0.76
rs950809		control	189	80(42.3)	80(42.3)	29(15.4)		0.37	
	ApoEε4(+)	AD	106	40(37.7)	58(54.7)	8(7.6)	0.29	0.35	0.61
		control	44	21(47.7)	18(40.9)	5(11.4)		0.32	

Abbreviations: MAF, minor allele frequency.

Genotypes and alleles are expressed as number (percentage). P values were calculated by χ^2^ test 3×2 contingency table for genotype distribution and 2×2 contingency table for allele distribution.

**Table 4:** Genotype and allele frequencies for rs17277986, rs6584777,rs10884402, rs7078098 and rs950809 stratified by gender in Alzheimer’s patients.

SNP ID	Group	n	Genotype frequency (%)			P-value	MAF	P-value
			CC	CT	TT		T	
rs17277986	AD(Male)	120	98 (81.7)	20 (16.7)	2 (1.6)	0.007	0.1	0.01
	AD(Female)	116	75 (64.7)	40 (34.5)	1 (0.8)		0.18	
			CC	CT	TT		T	
rs6584777	AD(Male)	120	98 (81.7)	20 (16.7)	2 (1.6)	0.007	0.1	0.01
	AD(Female)	116	75 (64.7)	40 (34.5)	1 (0.8)		0.18	
			GG	GA	AA		A	
rs10884402	AD(Male)	120	52 (43.3)	57 (47.5)	11 (9.2)	0.996	0.33	0.95
	AD(Female)	116	50 (43.1)	55 (47.4)	11 (9.5)		0.33	
			TT	TC	CC		C	
rs7078098	AD(Male)	120	62 (51.7)	52 (43.3)	6 (5.0)	0.295	0.27	0.25
	AD(Female)	116	55 (47.4)	49 (42.2)	12 (10.3)		0.31	
			CC	CT	TT		T	
rs950809	AD(Male)	120	44 (36.7)	64 (53.3)	12 (10)	0.991	0.37	0.92
	AD(Female)	116	43 (37.1)	62 (53.4)	11 (9.5)		0.36	

Abbreviations: MAF, minor allele frequency.

Genotypes and alleles are expressed as number (percentage). P values were calculated by χ^2^ test 3×2 contingency table for genotype distribution and 2×2 contingency table for allele distribution.

**Table 5:** Genotype and allele frequencies for rs17277986, rs6584777 stratified by gender in overall samples, Alzheimer’s patients and controls.

SNP ID	Group	n	Genotype frequency (%)			P-value	MAF	P-value
			CC	CT	TT		T	<0.0001
rs17277986	male	256	202(78.9)	52(20.3)	2(0.8)	0.003	0.09	
	female	213	138(64.8)	71(33.3)	4(1.9)		0.19	
			CC	CT	TT		T	<0.0001
rs6584777	male	256	203(79.3)	51(19.9)	2(0.8)	0.003	0.09	
	female	213	139(65.2)	70(32.9)	4(1.9)		0.18	
			CC	CT	TT		T	0.79
rs17277986	AD(Female)	116	75(64.6)	40(34.5)	1(0.9)	0.472	0.18	
	Control(Female)	97	63(64.9)	31(32)	3(3.1)		0.19	
			CC	CT	TT		T	0.9
rs6584777	AD(Female)	116	75(64.6)	40(34.5)	1(0.9)	0.446	0.18	
	Control(Female)	97	64(66)	30(30.9)	3(3.1)		0.19	
			CC	CT	TT		T	0.523
rs17277986	AD(Male)	120	98(81.7)	20(16.7)	2(1.6)	0.138	0.1	
	Control(Male)	136	104(76.5)	32(23.5)	0(0)		0.12	
			CC	CT	TT		T	0.61
rs6584777	AD(Male)	120	98(81.7)	20(16.7)	2(1.6)	0.163	0.1	
	Control(Male)	136	105(77.2)	31(22.8)	0(0)		0.11	

Abbreviations: MAF, minor allele frequency.

Genotypes and alleles are expressed as number (percentage). P values were calculated by χ^2^ test 3×2 contingency table for genotype distribution and 2×2 contingency table for allele distribution.

### Haplotype Analysis

**Figure 1:**
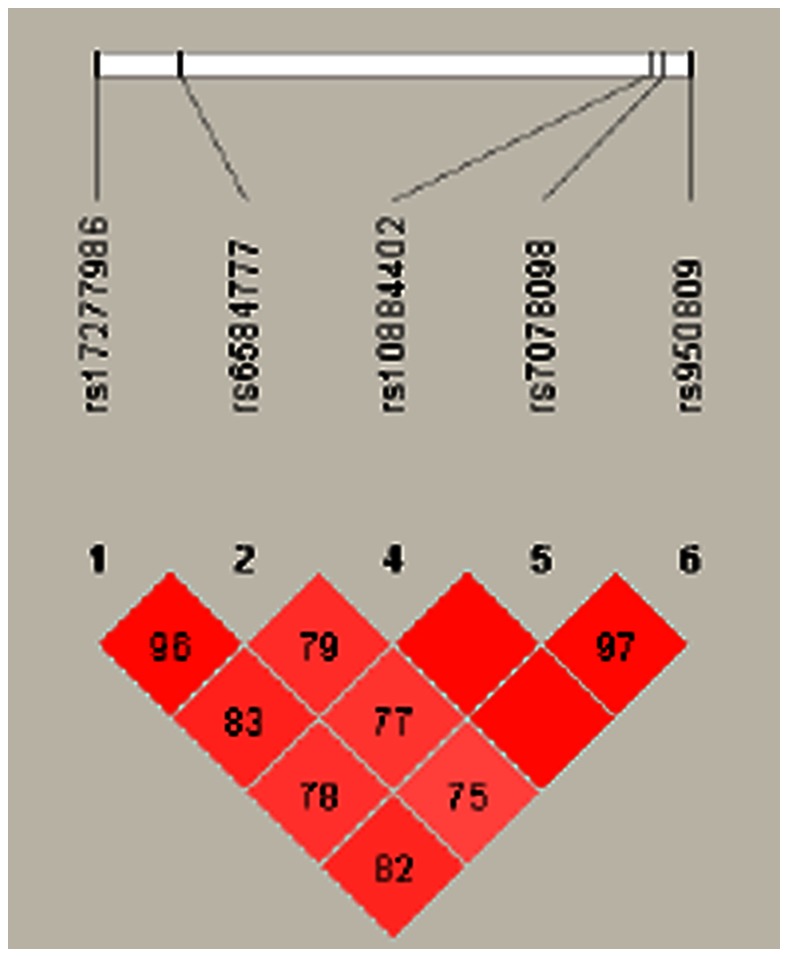
LD patterns of SNPs rs17277986,rs6585777, rs10884402, rs7078098 and rs950809. The linkage patterns between the 5 studied polymorphisms in all samples. The numbers in diamond represent the 100×D' in the form of standard color scheme. The upper bar denotes the relative distance among the studied polymorphisms. The bright red box denotes D' = 1 and LOD≥2; the pink box denotes D'<1 and LOD≥2.

Since the studied polymorphisms are assigned on the same chromosome, we accordingly performed the linkage analysis (Fig. 1), strong linkage patterns were observed between rs17277986 and rs6584777 in all samples (D' = 0.96), as well as among rs10884402, rs7078098 and rs950809 (D'≥0.97). Therefore, rs17277986 and rs6584777 constitute a block (block1) of two SNPs that are 5 kb apart, and rs10884402, rs7078098 and rs950809 constitute a block (block2) of three adjacent SNPs that are 2 kb apart and are in linkage disequilibrium (LD) in all samples. To facilitate identification of combinational effects of these five polymorphisms on AD risk, we employed haplotype analysis, which studies the frequency of the combination of multiple genetic variants. This is a more powerful statistical method than single-locus analysis. We focused on the haplotypes, which had a frequency of equal to or greater than 1% in all cases. The frequency of haplotypes composed of G-T-C (alleles in order of rs10884402, rs7078098, rs950809 polymorphisms, similarly hereinafter) was 34% in LOAD, which was significantly higher (Psim = 0.002) than that in control, whereas the frequencies of haplotypes composed of A-C-C and A-T-C was significantly lower (Psim<0.0001) in LOAD. Frequency of haplotype C-C-G-T-C (alleles in order of rs17277986, rs6584777, rs10884402, rs7078098, rs950809 polymorphisms, similarly hereinafter) was significantly higher (Psim = 0.0031), yet the frequencies of haplotype C-C-A-T-C, C-C-A-C-C and T-T-A-C-C were significantly lower (Psim<0.0001), in LOAD than in controls even after the statistical simulation (Table 6).

**Table 6:** Haplotype frequencies (>1%) of the five SNPs rs17277986, rs6584777, rs10884402, rs7078098 and rs950809 in SorCS1 gene and their relative risks for Alzheimer’s disease.

	Allele	All	LOAD	Control	Psim	OR (95% CI); P	OR (95% CI);P
	Combination	(n = 469)	(n = 236)	(n = 233)			(after ajustment^d^)
	1:C-C-A-T-C	0.03195	0	0.06212	<0.0001	NA	NA
	2:C-C-A-C-C	0.01804	0	0.03647	<0.0001	NA	NA
	3:T-T-A-C-C	0.01081	0	0.02143	<0.0001	NA	NA
Total^a^	4:C-C-A-T-T	0.31295	0.31208	0.32093	0.971	Reference	Reference
	5:T-T-G-C-C	0.10894	0.11189	0.10636	0.732	0.98(0.60,1.58);0.925	0.91(0.56,1.49);0.716
	6:C-C-G-T-T	0.0268	0.02873	0.02403	0.489	1.04(0.45,2.41);0.925	1(0.42,2.35);0.998
	7:C-C-G-C-C	0.16205	0.17556	0.14852	0.324	1.10(0.72,1.69);0.642	1.12(0.93,1.9);0.608
	8:C-C-G-T-C	0.29709	0.34099	0.2486	0.003	1.32(0.93,1.9);0.121	1.35(0.95,1.94);0.098
Block 1^b^	1:C-C	0.85608	0.86017	0.85193	0.708	Reference	Reference
	2:T-T	0.14179	0.13983	0.14378	0.916	0.96(0.65,1.4);0.815	0.91(0.62,1.33);0.617
	1:A-T-C	0.03203	NA	0.06496	<0.0001	NA	NA
	2:A-C-C	0.0289	NA	0.05759	<0.0001	NA	NA
Block 2^c^	3:A-T-T	0.32511	0.33051	0.32091	0.889	Reference	Reference
	4:G-C-C	0.2729	0.28724	0.25941	0.399	0.98(0.7,1.39);0.936	0.97(0.68,1.37);0.85
	5:G-T-T	0.02787	0.03088	0.02399	0.402	1.06(0.46,2.44);0.886	1.04(0.45,2.44);0.918
	6:G-T-C	0.30582	0.34836	0.26181	0.002	1.23(0.86,1.74);0.254	1.26(0.89,1.8);0.195

Abbreviations: OR, odds ratio; 95% CI, 95% confidence interval; Psim, Simulated P values; NA, not available.

a Alleles in total haplotype were arrayed in order of rs17277986, rs6584777, rs10884402, rs7078098 and rs950809.

b Alleles in block 1 haplotype were arrayed in order of rs17277986 and rs6584777.

c Alleles in block 2 haplotype were arrayed in order of rs10884402, rs7078098 and rs950809.

d Adjusting for age and gender.

## Discussion

APP processing and Aβ generation were considered to be the most important factors in pathogenesis of AD. Specifically, Small et al had suggested that APP processing might be modulated by Vps10-containing proteins [28], which could mediate the interaction between the retromer complex and APP. APP, β-secretases and γ-secretases were thus colocalized in the same intracellular compartment, where APP processing occurred. The genetic variations of *SORCS1*, the most recent member of the Vps10 family proteins [18], was also found to be associated with AD [20], [22], [29], [30] in Caribbean Hispanics, Caucasian Hispanics and et al. In our current study, rs10884402 and rs950809 in intron 1 of *SORCS1* were found to be associated with LOAD in Chinese Han population. The most noteworthy finding of this study was that rs10884402 showed significant difference between LOAD and the healthy controls in both genotype and haplotype analyses. The further stratified analysis also revealed the significant difference for rs10884402 polymorphism in both genotype and allele frequencies between the patients and controls in the ApoEε4 (−) population. The rs10884402 A allele displayed a significant protective effect against the risk of LOAD compared with the G allele in additive mode. The largerer number of A/A or A/G genotype in the ApoEε4 (−) normal control group reinforces our speculation that rs10884402 A allele is protective against AD. Moreover, we found this protective effect was irrelevant with the severity of AD. rs10884402 AA genotype vs G allele carriers confered a 67% decreased risk for LOAD in recessive mode. This conclusion in Chinese Han population was inconsistent with Reitz et al’s report that A allele of this SNP was associated with lower MMSE scores in Caribbean Hispanics [22]. Haplotype is composed of different alleles, thus haplotype analysis provides more information about the effect of genetic interaction on phenotype than single polymorphism analysis. Haplotype analyses in our study showed that haplotypes A-T-C and A-C-C (alleles in order of rs10884402, rs7078098, rs950809) were only observed in controls with total frequencies reaching 12%, whereas frequency of haplotype G-T-C was significantly higher in LOAD group than that in the controls, in agreement with the results of our single-locus analyses. However, this is also different from Reitz et al’s report that A-T-T haplotype for SNPs (alleles in order of rs10884402, rs7078098 and rs950809) were associated with LOAD in both NIA-LOAD dataset and Caribbean Hispanics datasets, and the complementary G-C-C haplotype was associated with higher MMSE scores in the NIA-LOAD dataset [22]. All above results indicated A allele in rs10884402 and C allele in rs950809 seemed to have a synergistic action because their combination was shown a protective effect against the risk of dementia, and A allele in rs10884402 might take a dominant place according to the results.

Our data showed negative association between rs17277986 and AD either in overall samples or in both gender subsets. However, when we reviewed all the case-control studies in the Alzgene data base and recent related studies, we found our result was different from that of Liang’s study [30], in which rs17277986 showed significant association with AD in the overall datasets (p = 0.0025) and in the female subset (allele p = 0.00002). However Liang et al could not confirm the association in their follow-up validation analyses in the validation datasets (CAP, the Collaborative Alzheimer Project; NCRAD, the NCRAD repository at Indiana University; NIMH, the National Institute of Mental Health repository) [30]. Our result was different from Reitz’s report either [30].

All above results indicated that several inconsistencies were presented in the different reports. However, we thought our data was reliable because of the following reasons. All SNPs in our research had been genotyped using the method of SNaPshot, which was an advanced and accurate gene analysis technique. All genotype distributions of SNPs in our research were conformed to the expected Hardy–Weinberg proportions. Moreover, the distributions of the APOE polymorphisms in AD patients and controls showed a significant difference, being the same as what was expected. And interestingly, our data about rs17277986 and rs6584777 are almost coincide with the results of the study in a Northern Han Chinese population conducted by Tan’s group [29], although the samples and experimental procedures were different. All these make our data trustworthy. One major and important reason for the inconsistency between our results and those of former studies is the different genetic background in different ethnic populations. The different environment and lifestyle may also need to be taken into consideration for the inconsistency because of the complexity of the interactions between genes and above factors. In addition, the size of sample group might be the third reason for the discrepancy. The larger sample size would be helpful for the further study.

In summary, our results implicated variations in intron 1 of *SORCS1* gene were associated with LOAD in Han Chinese. However, additional studies seeking to provide strong biological or clinical evidence for the association between *SORCS1* SNPs and LOAD, as well as longitudinal studies attempting to pursue gene–gene or gene–environment interactions of *SORCS1*, will be needed in the future.

## References

[1] Goedert M, Klug A, Crowther RA (2006) Tau protein, the paired helical filament and Alzheimer's disease. J Alzheimers Dis 9: 195–207. 10.3233/jad-2006-9s323 16914859

[2] Jellinger KA (2006) Alzheimer 100–highlights in the history of Alzheimer research. J Neural Transm 113: 1603–1623. 17039299 10.1007/s00702-006-0578-3

[3] Price DL, Sisodia SS (1998) Mutant genes in familial Alzheimer's disease and transgenic models. Annu Rev Neurosci 21: 479–505. 9530504 10.1146/annurev.neuro.21.1.479

[4] Gandy S (2005) The role of cerebral amyloid beta accumulation in common forms of Alzheimer disease. J Clin Invest 115: 1121–1129. 15864339 10.1172/JCI25100 PMC1087184

[5] Deng YL, Liu LH, Wang Y, Tang HD, Ren RJ, et al.. (2012) The prevalence of CD33 and MS4A6A variant in Chinese Han population with Alzheimer's disease. Hum Genet. 10.1007/s00439-012-1154-6 22382309

[6] Carrasquillo MM, Belbin O, Hunter TA, Ma L, Bisceglio GD, et al (2011) Replication of EPHA1 and CD33 associations with late-onset Alzheimer's disease: a multi-centre case-control study. Mol Neurodegener 6: 54. 21798052 10.1186/1750-1326-6-54 PMC3157442

[7] Gauthier S, Wu LY, Rosa-Neto P, Jia JP (2012) Prevention strategies for Alzheimer's disease. Translational Neurodegeneration 1: 13. 23210473 10.1186/2047-9158-1-13 PMC3514088

[8] Bertram L, Tanzi RE (2008) Thirty years of Alzheimer's disease genetics: the implications of systematic meta-analyses. Nat Rev Neurosci 9: 768–778. 18802446 10.1038/nrn2494

[9] Ashford JW, Mortimer JA (2002) Non-familial Alzheimer's disease is mainly due to genetic factors. J Alzheimers Dis 4: 169–177. 12226536 10.3233/jad-2002-4307

[10] Edbauer D, Winkler E, Regula JT, Pesold B, Steiner H, et al (2003) Reconstitution of gamma-secretase activity. Nat Cell Biol 5: 486–488. 12679784 10.1038/ncb960

[11] Xie ZC, Dong YL, Maeda U, Xia WM, Tanzi RE (2012) RNAi-mediated knock-down of Dab and Numb attenuate Aβ levels via γ-secretase mediated APP processing. Translational Neurodegeneration 1: 8. 23211096 10.1186/2047-9158-1-8 PMC3514095

[12] Nielsen MS, Gustafsen C, Madsen P, Nyengaard JR, Hermey G, et al (2007) Sorting by the cytoplasmic domain of the amyloid precursor protein binding receptor SorLA. Mol Cell Biol 27: 6842–6851. 17646382 10.1128/MCB.00815-07 PMC2099242

[13] Offe K, Dodson SE, Shoemaker JT, Fritz JJ, Gearing M, et al (2006) The lipoprotein receptor LR11 regulates amyloid beta production and amyloid precursor protein traffic in endosomal compartments. J Neurosci 26: 1596–1603. 16452683 10.1523/JNEUROSCI.4946-05.2006 PMC2638122

[14] Andersen OM, Reiche J, Schmidt V, Gotthardt M, Spoelgen R, et al (2005) Neuronal sorting protein-related receptor sorLA/LR11 regulates processing of the amyloid precursor protein. Proc Natl Acad Sci U S A 102: 13461–13466. 16174740 10.1073/pnas.0503689102 PMC1224625

[15] Hermey G, Riedel IB, Rezgaoui M, Westergaard UB, Schaller C, et al (2001) SorCS1, a member of the novel sorting receptor family, is localized in somata and dendrites of neurons throughout the murine brain. Neurosci Lett 313: 83–87. 11684345 10.1016/s0304-3940(01)02252-2

[16] Hermey G, Riedel IB, Hampe W, Schaller HC, Hermans-Borgmeyer I (1999) Identification and characterization of SorCS, a third member of a novel receptor family. Biochem Biophys Res Commun 266: 347–351. 10600506 10.1006/bbrc.1999.1822

[17] Jacobsen L, Madsen P, Moestrup SK, Lund AH, Tommerup N, et al (1996) Molecular characterization of a novel human hybrid-type receptor that binds the alpha2-macroglobulin receptor-associated protein. J Biol Chem 271: 31379–31383. 8940146 10.1074/jbc.271.49.31379

[18] Hermey G, Plath N, Hubner CA, Kuhl D, Schaller HC, et al (2004) The three sorCS genes are differentially expressed and regulated by synaptic activity. J Neurochem 88: 1470–1476. 15009648 10.1046/j.1471-4159.2004.02286.x

[19] Lane RF, Gatson JW, Small SA, Ehrlich ME, Gandy S (2010) Protein kinase C and rho activated coiled coil protein kinase 2 (ROCK2) modulate Alzheimer's APP metabolism and phosphorylation of the Vps10-domain protein, SorL1. Mol Neurodegener 5: 62. 21192821 10.1186/1750-1326-5-62 PMC3036620

[20] Reitz C, Tokuhiro S, Clark LN, Conrad C, Vonsattel JP, et al (2011) SORCS1 alters amyloid precursor protein processing and variants may increase Alzheimer's disease risk. Ann Neurol 69: 47–64. 21280075 10.1002/ana.22308 PMC3086759

[21] Laumet G, Chouraki V, Grenier-Boley B, Legry V, Heath S, et al (2010) Systematic analysis of candidate genes for Alzheimer's disease in a French, genome-wide association study. Journal of Alzheimer's disease : JAD 20: 1181–1188. 20413850 10.3233/JAD-2010-100126

[22] Reitz C, Lee JH, Rogers RS, Mayeux R (2011) Impact of genetic variation in SORCS1 on memory retention. PLoS One 6: e24588. 22046233 10.1371/journal.pone.0024588 PMC3202519

[23] Dubois B, Feldman HH, Jacova C, Dekosky ST, Barberger-Gateau P, et al (2007) Research criteria for the diagnosis of Alzheimer's disease: revising the NINCDS-ADRDA criteria. Lancet Neurol 6: 734–746. 17616482 10.1016/S1474-4422(07)70178-3

[24] Corder EH, Saunders AM, Strittmatter WJ, Schmechel DE, Gaskell PC, et al (1993) Gene dose of apolipoprotein E type 4 allele and the risk of Alzheimer's disease in late onset families. Science 261: 921–923. 8346443 10.1126/science.8346443

[25] Lake SL, Lyon H, Tantisira K, Silverman EK, Weiss ST, et al (2003) Estimation and tests of haplotype-environment interaction when linkage phase is ambiguous. Hum Hered 55: 56–65. 12890927 10.1159/000071811

[26] Stram DO, Leigh Pearce C, Bretsky P, Freedman M, Hirschhorn JN, et al (2003) Modeling and E-M estimation of haplotype-specific relative risks from genotype data for a case-control study of unrelated individuals. Hum Hered 55: 179–190. 14566096 10.1159/000073202

[27] Schaid DJ, Rowland CM, Tines DE, Jacobson RM, Poland GA (2002) Score tests for association between traits and haplotypes when linkage phase is ambiguous. Am J Hum Genet 70: 425–434. 11791212 10.1086/338688 PMC384917

[28] Small SA, Gandy S (2006) Sorting through the cell biology of Alzheimer's disease: intracellular pathways to pathogenesis. Neuron 52: 15–31. 17015224 10.1016/j.neuron.2006.09.001 PMC4820242

[29] Wang HF, Yu JT, Zhang W, Wang W, Liu QY, et al (2012) SORCS1 and APOE polymorphisms interact to confer risk for late-onset Alzheimer's disease in a Northern Han Chinese population. Brain Res 1448: 111–116. 22353753 10.1016/j.brainres.2012.01.067

[30] Liang X, Slifer M, Martin ER, Schnetz-Boutaud N, Bartlett J, et al (2009) Genomic convergence to identify candidate genes for Alzheimer disease on chromosome 10. Human mutation 30: 463–471. 19241460 10.1002/humu.20953 PMC2713862

